# BMAL1 in the Adrenal Gland: It’s About Time—A Perspective on “Adrenal-Specific KO of the Circadian Clock Protein BMAL1 Alters Blood Pressure Rhythm and Timing of Eating Behavior”

**DOI:** 10.1093/function/zqad008

**Published:** 2023-02-16

**Authors:** Brittni N Moore, Jennifer L Pluznick

**Affiliations:** Department of Physiology, Johns Hopkins University School of Medicine 725 N, Wolfe St, WBSB 205 Baltimore, MD 21205, USA; Department of Physiology, Johns Hopkins University School of Medicine 725 N, Wolfe St, WBSB 205 Baltimore, MD 21205, USA

**Keywords:** BMAL1, adrenal gland, perspective, adrenal, specific, KO

The circadian clock is always ticking, working to synchronize both our behaviors and our internal physiology to the rhythms of the outside world. In mammals, the circadian clock can be divided into two systems: the core clock, located in the hypothalamic superchiasmatic nucleus, and the peripheral clocks, which are cell autonomous and operate in nearly every tissue.^1^ Both systems regulate biological processes, and rely on cues known as zeitgebers for entrainment.^2^ On a molecular level, the biological clock consists of core proteins that work together in a feedback loop to regulate the circadian rhythm.^1^ The positive arm of this loop consists of the heterodimer, BMAL1:CLOCK, which activates transcription of Per and Cry genes.^3^ The negative arm comprises the protein products of the positive regulatory loop, CRY and PER, which repress BMAL1 and CLOCK activity, and thus suppress their own transcription.^3^ Mutations in clock genes can disrupt sleeping behaviors and contribute to disease.^3^

Blood pressure exhibits a strong circadian rhythm, including a “dip” during the resting period and a surge upon waking; loss of this pattern is associated with cardiovascular disease. Of note, whole animal knockout (KO) animals for PER1,^4^ CRY1/CRY2,^5^ and BMAL1^6^ display blood pressure phenotypes. In recent years, it has become apparent that the *peripheral* circadian clocks also play a role in blood pressure regulation. Altered blood pressure and/or vascular function occurs when clock genes are deleted in smooth muscle, perivascular adipose, and various segments of the renal nephron.^7^ Of note, the phenotype associated with whole-animal BMAL1 KOs is not fully replicated in any one tissue-specific KO. Intriguingly, there are hints in the literature that the adrenal gland may play a role in circadian rhythms in blood pressure, for example, double Cry1/2 KO mice have an adrenal disorder,^5^ and adrenal clock genes are phase advanced in spontaneously hypertensive rats.^8^ In addition, the adrenal gland synthesizes cortisol and aldosterone, both of which influence blood pressure regulation. Thus, it is tempting to hypothesize that the adrenal gland clock may contribute to blood pressure rhythmicity. However, until recently, a role for the peripheral clock in the adrenal gland had not been systematically examined.

In a timely investigation, Costello et al. generated an adrenal-specific BMAL1 KO mouse to study the influence of the adrenal gland on blood pressure regulation and blood pressure rhythms.^9^ Given that female kidney-specific BMAL1 KO mice do not exhibit changes in blood pressure or blood pressure rhythms, Costello et al. focused on males. This study found that KO of BMAL1 in the zona glomerulosa of the adrenal cortex resulted in a shortened blood pressure and activity period, and a delayed peak of both blood pressure and activity. These findings are particularly impressive because they were present under normal light:dark conditions; typically, circadian rhythm defects require constant conditions in order to be detected.

In an unexpected finding, the investigators did not observe blood pressure and activity changes in mice housed in home cages, but did find significant changes in blood pressure rhythm when mice were housed in metabolic cages. Mice housed in metabolic cages exhibited increased serum corticosterone (the mouse equivalent of cortisol), and thus the authors concluded that metabolic cages acted as a mild stressor. Mice in metabolic cages also exhibited blunted night/day differences in serum concentrations of corticosterone. This clever finding of home cage versus metabolic cage differences has important implications for all studies that use metabolic cages, and is an important reminder to consider how housing and other environmental factors may unexpectedly influence phenotypes.

Given that food intake both exhibits a circadian rhythm and is a powerful zeitgeber for peripheral clocks,^10^ Costello and team explored whether eating behaviors in KO mice contributed to these phenotypes. Investigators found no significant differences in total food consumption between KO and WT mice; however, they discovered night:day food intake was significantly blunted in KO males compared to littermate controls.

In sum, this work by Costello et al. utilized well-designed and controlled experiments to uncover the role of adrenal BMAL1 on blood pressure regulation and rhythmicity for the first time. This study has expanded our knowledge of the circadian clocks to include a role for the adrenal gland; as noted above, the phenotype in the adrenal KO mice is particularly impressive in that it is apparent in normal light:dark conditions. This clearly presented study provides valuable insight into how the adrenal gland and the molecular clock intersect to regulate blood pressure.

Like all circadian studies, this study also serves as a reminder of the importance of controlling for “time of day” as a variable. And like all impactful findings, this study begs additional questions: Are alterations in eating patterns, activity, and blood pressure compensatory in these KO models, or maladaptive? What are the relative impacts of the various peripheral clocks, in comparison to the central clock? How do clock proteins operate in males versus females, and what governs sex differences in clock protein function? How are these clocks altered in disease? How quickly can clocks “adapt” to shift work? Clearly, future studies are needed to answer these questions, and to more fully understand the impactful role of the circadian clock in physiology. Although it is tempting to speculate as to what these future studies will uncover, only time will tell.
